# Acquired Resistance to PD-1/PD-L1 Blockade in Lung Cancer: Mechanisms and Patterns of Failure

**DOI:** 10.3390/cancers12123851

**Published:** 2020-12-20

**Authors:** Ranjan Pathak, Rebecca R. Pharaon, Atish Mohanty, Victoria M. Villaflor, Ravi Salgia, Erminia Massarelli

**Affiliations:** Department of Medical Oncology and Therapeutics Research, City of Hope National Medical Center, Duarte, CA 91010, USA; rpathak@coh.org (R.P.); rpharaon@coh.org (R.R.P.); amohanty@coh.org (A.M.); vvillaflor@coh.org (V.M.V.); rsalgia@coh.org (R.S.)

**Keywords:** lung cancer, PD-1, PD-L1, immunotherapy, immune checkpoint inhibitors, acquired resistance

## Abstract

**Simple Summary:**

Acquired resistance to immunotherapy is an emerging issue, especially as the indications for immunotherapy expand to many different solid tumor malignancies. This review attempts to summarize the mechanisms and clinical outcomes of acquired resistance in non-small-cell lung cancers and explores future directions for research to understand and address this important question.

**Abstract:**

Immunotherapy is now the preferred treatment for most lung cancer patients. It is used to treat unresectable stage III non-small-cell lung cancer and is the first-line therapy for non-oncogene-driven advanced/metastatic non-small-cell lung cancer patients (either alone or in combination with chemotherapy). Unfortunately, most patients that respond initially to immunotherapy develop resistance over time, thus limiting the durability of immunotherapy. A better understanding of the mechanisms of acquired resistance is urgently needed to expand the benefit of immunotherapy in lung cancer patients. This review aims to summarize the mechanisms and clinical outcomes of acquired resistance of anti-PD-1/PD-L1 therapies in non-small-cell lung cancer patients.

## 1. Introduction

**Figure 1:**
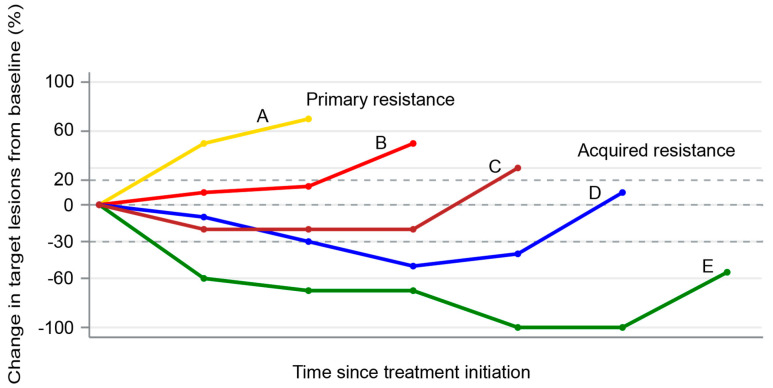
Spider plot showing examples of primary (patient A) and acquired resistance (patients B–D) to immune checkpoint inhibitors. Patient A represents disease progression as the best response. Patients B and C represent initial stable disease followed by progression. Patient D represents an initial partial response followed by disease progression. Patient E represents complete response followed by disease progression.

Immunotherapy represents a new paradigm for the treatment of non-small-cell lung cancers (NSCLC), with immune checkpoint inhibitors (ICI) such as anti-programmed death-1 (PD-1)/PD-ligand-1 (PD-L1) leading the field. The binding of PD-1 to its ligands (PD-L1 or PD-L2) on tumor cells suppresses T cells through a negative feedback loop, thereby leading to evasion of the immune response [1,2]. Although antibodies targeting PD-1 or PD-L1 have demonstrated impressive and durable clinical efficacy in a subset of patients with NSCLC, many patients do not respond at all (primary resistance), whilst others that initially respond ultimately relapse (acquired resistance) (Figure 1) [3].

Limited knowledge exists on the mechanisms mediating resistance to ICIs in lung cancer. Recent research has demonstrated that some genomic alterations might confer primary resistance to ICIs, including STK11 and KEAP1 mutations [4,5]. Similarly, increased activity of enhancer of zeste homolog 2 (EZH2) has been associated with acquired resistance to ICI in NSCLC [6]. Other mechanisms, such as clonal selection of tumor cells lacking neoantigens [7], low non-synonymous tumor burden [8], and impaired HLA class I antigen function, have been described in lung cancer [9]. However, the majority of our understanding of ICI resistance comes from research done on melanoma. Although it is plausible that lung cancers share many of the pathways leading to ICI resistance with solid tumor cancers, further research is needed to clarify this. A precise understanding of the molecular underpinnings of ICI resistance pathways is needed to develop rational combination therapies that can overcome acquired ICI resistance.

In this review, we aim to explore the mechanisms of acquired ICI resistance in lung cancer, describe the patterns of failures in patients with acquired resistance, and discuss areas for future research.

## 2. PD-1/PD-L1 Axis in Normal Health and Tumorigenesis

The development of a naïve T cell into an effector cell involves activation of T cells in secondary lymphoid structures (such as lymph nodes) and involves three distinct signals: T cell receptor (TCR) engagement of peptide-major histocompatibility complex (MHC) (signal 1), co-stimulation between CD28 and B7-1 (CD80) or B7-2 (CD86) (signal 2), and inflammatory cytokines (such as IL-12 and type 1 interferon (IFN)) (signal 3) [10]. PD-1 is a crucial inhibitory receptor which is mainly present on activated T cells and is one of the many receptors that negatively regulate T cell activation (so-called immune checkpoints). While it is also important for fighting infections and mediating tissue repair, PD-1/PD-L1 and other checkpoint signaling is crucial for maintaining the balance between positive and negative regulation of T cells, maintaining immune tolerance, preventing autoimmunity, and avoiding excessive immune response [11].

Activated T cells produce inflammatory cytokines such as IFN-γ that lead to upregulation of PD-L1 on the antigen-presenting cells [12]. Engagement of PD-1 with PD-L1 leads to dampening of the TCR-mediated T cell activation. In addition, PD-1/D-L1 interaction leads to inhibition of the CD28-mediated co-stimulation [13]. PD-L1 can also compete with CD80 to bind to CD28, leading to further inhibition of the T cell response [14].

Despite producing numerous neoantigens (such as proteins encoded by the mutated genes [15] or aberrant transcripts produced in the dysregulated cancer cell nucleus [16]), cancer cells are often able to evade immune detection and immune-mediated killing by several mechanisms [17]. Early experiments showed that in mouse models of myeloma, tumors were found to upregulate surface PD-L1 to reduce T cell effector functions, and the growth of transplanted tumor cells could be reduced by using anti-PD-L1 antibodies [2]. Observations such as these [18,19,20], along with the findings of upregulation of PD-L1 in a variety of different solid tumors [21], led to further research and development of anti-PD-1/PD-L1 antibodies in humans. As of 2020, we now have four FDA-approved anti-PD-1 and anti-PD-L1 therapies for lung cancers alone. 

## 3. Mechanisms of Acquired ICI Resistance

**Figure 2:**
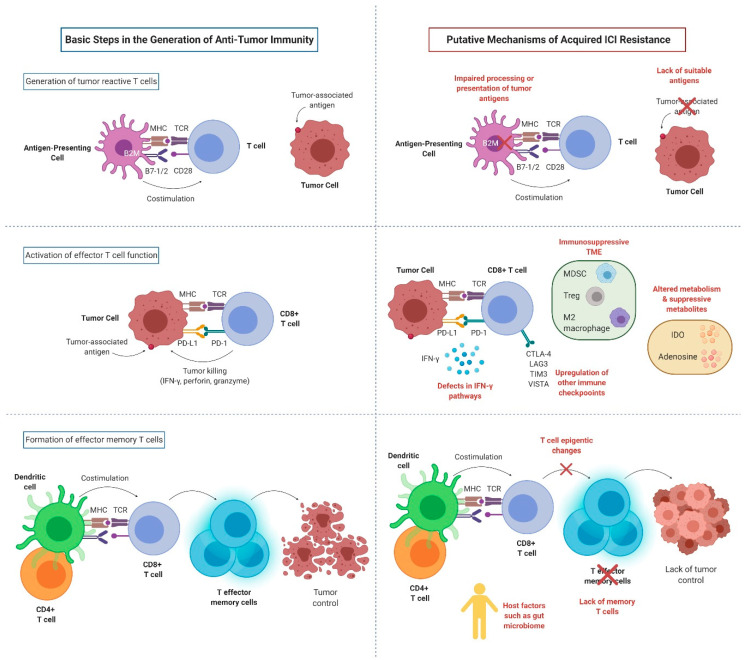
Key mechanisms of acquired resistance to anti-PD-1/PD-L1 inhibitors (created with BioRender.com). MHC: major histocompatibility complex; TCR: T cell receptor; CD28: cluster of differentiation 28; B7-1: cluster of differentiation 80; B7-2: cluster of differentiation 86; B2M: B2-microglobulin; PD-1: programmed cell death protein 1; PD-L1: programmed death-ligand 1; IFN-γ: interferon gamma; CTLA-4: cytotoxic T-lymphocyte-associated protein 4; LAG3: lymphocyte-activation gene 3; TIM3: T cell immunoglobulin and mucin domain-containing molecule 3; VISTA: V-domain Ig suppressor of T cell activation; MDSC: myeloid-derived suppressor cells; Treg: regulatory T cell; IDO: indoleamine 2,3-dioxygenase.

For anti-PD-1/PD-L1 therapies to be effective, several conditions need to be satisfied: (1) a sufficiently immunogenic tumor; (2) efficient activation and infiltration of T cells to the tumor microenvironment (TME); (3) tumor dependence on the PD-1/PD-L1 axis for evasion of the anti-tumor immune responses; (4) sufficient reinvigoration of exhausted tumor-specific CD8 + T cells; (5) T cell specificity for a wide variety of tumor antigens or for proteins essential for tumor growth, and display of these antigens on MHC on the surface of tumor cells; and (6) generation of long-lived effector T cells or memory T cells (durable remission). Tumors can exploit any of these steps (alone or in combinations) to acquire resistance to ICIs (Figure 2). 

### 3.1. Impaired T Cell Activation

#### 3.1.1. Loss of Antigen Presentation 

Acquired resistance to ICI can be attributed to ineffective display of antigens, along with MHC molecules. Effective T cell-mediated response to ICI entails expression and recognition of antigenic peptides. Tumor cells can downregulate the expression of MHC–antigen complexes to prevent recognition by effector T cells [22]. Immunoediting during tumorigenesis can lead to the development of tumor clones that have low MHC expression [23] or have neoantigens that bind poorly to MHC [24], and this has been shown in the context of ICI resistance. Anagnostou et al. showed that selective pressure of ICI can lead to selection of tumor clones that have lost mutations associated with neoantigen generation, leading to acquired resistance to ICI in NSCLC [7]. In addition, Gettinger et al. showed that acquired resistance to PD-1 axis inhibitor therapy in NSCLC can arise due to acquired homozygous loss of B2-microglobulin (B2M, a protein vital for antigen presentation by MHC class I)*,* which led to a lack MHC class 1 expression in the cell surface of the tumor and a matched patient-derived xenograft [9].

#### 3.1.2. Fixed Epigenetic Modification and Lack of Memory T Cells

PD-1 blockade can reinvigorate hypofunctional “exhausted” CD8 + T cells (Tex), restoring their anti-tumor effector functionality [25]. Tumor-infiltrating Tex cells are characterized by increasing dysfunction driven by chromatin remodeling and epigenetic modifications, eventually reaching a state of fixed epigenetic dysfunction in which their chromatin is rendered inaccessible and is thus resistant to further remodeling and reinvigoration [26]. If the tumor burden remains high and reinvigorated, Tex cells fail to eradicate the tumor cells, and they eventually become re-exhausted and resistant to reinvigoration by anti-PD-1 therapy [27].

Although studies in NSCLC are lacking, studies examining melanoma patients suggest that the expansion of intratumoral memory T cells might be associated with the efficacy of anti-PD-1 therapy [28]. This suggests that impaired generation of memory T cells might lead to a waning of the efficacy of PD-1/PD-L1 blockade over time, resulting in acquired resistance [3]. Taken together, these studies suggest that a lack of memory T cells and re-exhausted Tex cells might lead to acquired resistance to PD-1 blockade [28].

### 3.2. Impaired T Cell Effector Functions

#### 3.2.1. Defects of Interferon Pathways 

While mutations within IFN signaling elements have been described in the setting of primary resistance to ICIs, these mutations can also develop following PD-1/PD-L1 blockade. IFN-γ leads to an upregulation of PD-L1 expression in tumors, increased antigen production, and increased release of T cell-attracting chemokines [29]. JAK1 or JAK2 inactivating mutations have been described in melanoma patients that prevent IFN-γ signaling, PD-L1 expression, and acquired resistance to anti-PD-1 therapy [30]. In addition, deletion of genes necessary for IFN-γ signaling (such as IFNGR1, IFNGR2, STAT1, JAK1, and JAK2) have been noted in pre-clinical models of melanoma that can lead to resistance to anti-PD-1/PD-L1 therapies, which might also play a role in acquired resistance [31].

#### 3.2.2. Upregulation of Other Immune Checkpoints

Expression of other immune checkpoint molecules, including CTLA-4, T cell immunoglobulin and mucin domain-containing molecule-3 (TIM-3), lymphocyte activation gene-3 (LAG-3), and V-domain Ig suppressor of T cell activation (VISTA), etc. [32] has been proposed to lead to acquired resistance to PD-1/PD-L1 blockade. LAG-3 has structural homology to CD4 and competes for binding to MHC class II, which leads to decreased efficacy of MHC class II-mediated antigen presentation. Johnson et al. found that melanoma and lung cancer patients with acquired resistance to anti-PD-1therapy demonstrated upregulation of LAG-3 in their tumors [33]. TIM-3 is an inhibitory receptor expressed on IFN-γ producing CD4 + Th1 and CD8 + T cells. PD-1 and TIM-3 co-expression has been reported in severely exhausted T cells [34]. In immunocompetent mouse models of lung adenocarcinoma, TIM-3 has been found to be upregulated following PD-1 blockade with anti-TIM-3 antibody, resulting in a survival advantage [35].

#### 3.2.3. Altered Metabolism and Suppressive Metabolites

Recent studies have shown that tumor cells develop an altered metabolism to allow for sustained, uncontrolled growth in a nutrient-poor environment [36,37]. Tumor cells can outcompete T cells for glucose uptake to reduce glycolytic activity and IFN-γ production by T cells [36]. In pre-treatment melanoma tumors, hypoxia-associated genes are highly expressed in the tumors that are subsequently resistant to PD-1 blockade compared with those from responding tumors [37].

Similar to PD-L1, indoleamine 2,3-dioxygenase (IDO) expression can also be influenced by IFN-γ, and can lead to primary resistance to PD-1 blockade [38]. IDO is the initial and rate-limiting enzyme in the degradation of tryptophan through the kynurenine pathway. This decrease in tryptophan can inhibit the activation and function of effector T cells, and increase attraction and activation of pre-existing Tregs, as well as the differentiation of naïve T cells into Tregs [39]. Studies have shown that NSCLC patients with early progression on nivolumab have a significantly higher kynurenine/tryptophan ratio, suggesting that IDO might contribute to primary resistance to anti-PD-1/PD-L1 monoclonal antibodies [40].

Adenosine is an immunosuppressive molecule that can suppress effector T cells and increase Treg numbers in addition to suppressing NK cell maturation and function via the A2a receptor [41,42]. Tregs also express ectoenzymes CD39 and CD73, which metabolize ATP to adenosine [43]. Similarly CD38, CD73, and CD203a ectoenzymatic pathways can metabolize NAD+ to adenosine [44]. Adenosine deaminase (ADA) degrades adenosine when bound to the cell surface receptor CD26 [43]. In a NSCLC mouse model, CD38 was found to be upregulated on tumor cells upon resistance to anti-PD-1 axis therapy, which could be reversed with the inhibition of either A2a or CD38 [45]. Based on the observation of reduction of PD-1, TIM-3, and LAG-3 expression on effector CD8 + T cells and Tregs after the blockade of A2a receptor in mice, researchers suggested that decreased PD-1 expression with A2a inhibitor might lessen the threshold for an effective anti-PD-1 axis therapy [46].

### 3.3. Other Local Immunosuppressive Factors within the TME

In addition to the above mechanisms, the influx of immunosuppressive cell populations such as Tregs, myeloid-derived suppressor cells (MDSC), and M2 polarized macrophages into the TME can itself lead to decreased effector T cell effector functions in patients receiving PD-1 blockade therapy [47]. MDSCs represent a heterogeneous population of immature myeloid cells that can result in acquired resistance to ICI via various mechanisms [48,49]. Similarly, Tregs, which are a subtype of CD4 + T cells, can suppress the activity and proliferation of CD8 + T cells, leading to acquired ICI resistance [50]. In a study involving mouse models of head and neck squamous cell cancers, acquired resistance to PD-1 blockade was associated with increased intratumoral Treg infiltration [51]. Moreover, several cytokines, such as CCL5, CCL17, CCL22, CXCL8, and CXCL12, transforming growth factor beta (TGF-β), and vascular endothelial growth factor (VEGF), have been shown to be associated with ICI resistance in various tumors [52,53]. Studies examining the impact of these immunosuppressive TME components in NSCLC are needed to understand the impact of these factors in the development of acquired resistance to PD-1/PD-L1 blockade in lung cancer.

### 3.4. Role of Host Factors in ICI Resistance

Of the various putative host factors, gut microbiome has aroused the most interest in the recent years. Several studies have suggested that the gut microbiome might influence anti-tumor responses by modulating both innate and adaptive immunity [54,55]. For example, a study by Routy et al. found a positive correlation between the relative abundance of *Akkermansia muciniphila* and the efficacy of ICI, and oral supplementation with Akkermansia restored the activity of anti-PD-1 therapy in mice [56]. In another study involving melanoma patients, anti-PD-1 axis therapy was found to have an increased gut *Ruminococcaceae* in responders [57].

Along this line, the use of antibiotics in patients receiving ICI has also been studied extensively, given the concerns about antibiotics compromising the efficacy of ICIs by altering gut microbiome [58,59,60]. A recent study by Pinato et al. demonstrated that prior antibiotic use (but not concurrent antibiotic use) was associated with a poor response to ICI therapy and a worse overall survival independent of the type of antibiotics, performance status, and steroids [61]. The authors hypothesized that antibiotics perhaps lead to a prolonged disruption of the gut microbiome, thus impairing the effectiveness of anti-PD-1 axis therapy. Some preliminary data have suggested that microbiota might influence anti-tumor immunity through gut metabolites (such as anacardic acids), facilitating T helper cell response and maturation of dendritic cells [62]. Although these are interesting observations, further studies are needed to elucidate the exact mechanisms of this association and the impact of the type and duration of antibiotics on survival in these patients.

While it is still debated, some evidence suggests that baseline corticosteroid use of ≥10 mg prednisone equivalent is associated with inferior outcomes in NSCLC patients treated with ICIs [63,64]. Although it is unclear if the poorer outcomes seen in these patients are directly related to the immunosuppressive effect of corticosteroid use versus a reflection of selection bias (e.g., patients with symptomatic brain metastases, steroid use for palliative reasons), it is probably wise to use corticosteroids judiciously until larger confirmatory studies are available.

## 4. Patterns of Failures After PD-1/PD-L1 Blockade

Studies examining the patterns of failures in NSCLC after PD-1/PD-L1 blockade are sparse. Difficulty in assessing progression on ICI has led to some describing acquired resistance as disease progression after partial response or complete response (CR) [65], while others have described it as initial clinical benefit followed by the development of progression [66]. Widespread use of immune-related response criteria (iRECIST) [67], which requires additional follow-up scans to confirm or refute an “unconfirmed progressive disease” designation, will hopefully harmonize the definitions of acquired resistance and lower erroneous terminations of ICI therapy. Novel methods such as circulating tumor DNA [68], TCR sequencing on peripheral T cells [69], tumor biopsies immune-profiling analyses by next-generation sequencing and high-throughput flow cytometry/mass cytometry [70], and isotope-labeled PD-1 and PD-L1 targeted imaging [71] will hopefully lead to a more accurate and reliable diagnosis of acquired ICI resistance in the future.

Patterns of failures after ICI can either be in new sites or established sites. In patients who relapse after a CR, many seem to develop new sites of metastasis, while the initial disease is controlled. In a study of ICI-treated (the majority with anti-PD-1/PD-L1 blockade) melanoma patients (*N* = 428), 77 patients achieved an ongoing CR, while 351 experienced progressive disease. Of the patients who survived after an incomplete response or progressive disease to ICI treatment (*N* = 282), 52 were treated with local therapy to control oligoprogression outside of the brain. Seven out of the eight patients (88%) who recurred after a CR recurred in a new site, which suggests that ICI can still fail to clear occult micrometastases, which can progress later as new sites of disease [72]. In contrast, progression at sites of established disease suggests that ICI was effective in clearing clinically occult micrometastases but was associated with local immune failure, likely from the development of an immune escape mechanism. Although limited by small sample size, the authors reported that the melanoma patients who achieved no evidence of disease after local therapy had significantly longer progression-free survival (PFS)when progression was limited to established tumors (*N* = 15) compared with patients who developed new lesions (*N* = 22) [72]. Similar results were seen in another study of melanoma patients treated with combined anti-PD-1 and anti-CTLA-4 therapy, with the majority of patients progressing after CR doing so in new sites versus established sites [73].

Only a handful of studies have reported the clinical outcomes of progression on PD-1 blockade therapy in NSCLC. In a study involving 34 NSCLC patients who developed acquired resistance to PD-1 axis blockade therapy, factors significantly associated with PFS were performance status and the depth of response to immunotherapy on multivariable analyses [66]. A majority of patients (61%) had progression of their existing disease rather than development of new lesions. About 2/3 of the patients progressed at a single organ, while the rest had multi-site progression. A majority of the patients (82%) had progressive disease as their best reported response to ICI [66].

In another study by Gettinger et al., 26 patients with acquired resistance to PD-1 axis inhibitor therapy were evaluated [65]. The most common site of progression was in the lymph nodes (77%). Recurrence was limited to one or two sites of disease in 88% of patients. The authors concluded that continuation of PD-1 therapy with local therapy can achieve prolonged benefit in patients with 1–2 sites of disease [65]. This observation needs to be validated in larger studies, but it is possible that prior therapy might alter the immune microenvironment or render tumors more immunogenic due to immune editing in the presence of prior chemotherapy.

## 5. Principles of Overcoming Acquired Resistance to PD-1/PD-L1 Blockade

Improved understanding of the underlying acquired resistance mechanisms is crucial to develop strategies to combat resistance. One can argue that ICI perhaps selectively kills only the intrinsically sensitive tumor cells, thus leaving resistant clones to persist and eventually proliferate. Mechanistic studies are thus needed to understand the highly complex and heterogeneous TME compartments that might have a differential sensitivity to T cell-mediated killing [74]. Combination therapies (such as with chemotherapy) that eliminate multiple tumor subclones from the outset can potentially prevent resistance due to the persistence of resistance clones. Another possibility for the development of acquired resistance is the immune editing and evolution of tumor and immune cells under the pressure of PD-1/PD-L1 blockade that may transform sensitive tumors into resistance tumors (as discussed in earlier sections).

Attempts to study these mechanisms will likely require the collection of sequential pre-treatment, on-treatment, and post-relapse tissue and blood samples. Pre-treatment biopsies could pave the way for biomarker-based adaptive therapy. Based on the genetic and immunological profile of the pre-treatment biopsy, either ICI alone or a “lead-in” therapy with a sensitizing agent could be instituted. Sensitizing agents could be further modified based on temporal changes on the immunological profiles of sequential biopsies and peripheral blood sampling. Evaluation of temporal changes in TME, peripheral blood chemokines, and immunological profiles has the potential to help to design adaptive therapies that can overcome acquired resistance to ICIs in an efficient way. Application of modern systems biology techniques such as computational systems biology can help to integrate the complex information gathered from tumor biopsies and peripheral blood samples into the design of effective therapeutics [75].

Various combinatorial approaches utilizing cytokines, tumor-infiltrating lymphocytes, chimeric antigen receptor T cell therapy, vaccines, chemotherapy, as well as other ICIs are currently under investigation to help to overcome resistance to PD-1 axis blockade [52]. Although an in-depth discussion of these therapeutic strategies is outside the scope of this review, we have provided a brief summary of potential therapeutic approaches to overcome ICI resistance in NSCLC (Table 1).

## 6. Conclusions and Future Directions

As of today, numerous PD-1 axis inhibitors are approved in locally advanced and metastatic NSCLC including, but not limited to, pembrolizumab, nivolumab, durvalumab, and atezolizumab. While these approvals have radically transformed the treatment landscape of NSCLC with durable anti-tumor responses and an unprecedented 16% five-year survival rate among patients with pre-treated advanced NSCLC, much remains unknown about the mechanisms of resistance. Carefully designed mechanistic studies, including sequential tumor biopsies and peripheral blood sampling throughout the course of treatment, have the potential to detect mechanisms of acquired resistance on ICI. Use of computational systems biology techniques might enable integration of complex information derived from genomics, epigenetics, immunology, proteomics, etc. to design effective strategies to overcome acquired ICI resistance and expand the reach of anti-PD-1/PD-L1 agents to a greater number of NSCLC patients.

## Figures and Tables

**Table 1:** Putative mechanisms and approaches to overcome acquired immune checkpoint inhibitor (ICI) resistance in non-small-cell lung cancer. Most approaches have multiple potential mechanisms of actions.

Mechanisms of Acquired ICI Resistance	Potential Therapeutic Approaches
Impaired processing or presentation of tumor-associated antigens	Chemotherapy, radiotherapy, cancer vaccines, oncolytic viruses, TLR agonists
Fixed epigenetic modification of T cells	Epigenetic modulators (hypomethylating agents, histone deacetylase inhibitors)
Defects in IFN-γ pathways	STING agonists, c-di-GMP, JAK inhibitors
Upregulation of other immune checkpoints	Combination immune checkpoint inhibitors (such as CTLA-4, TIGIT, LAG-3, TIM-3, VISTA, BTLA), immune stimulatory agents (such as OX40, CD40, GITR, ICOS, 4-1BB)
Immunosuppressive cells/inhibitory metabolites in the tumor microenvironment	Macrophage inhibitors (such as CSF1R inhibitors), cytokine/chemokine inhibitors, targeted therapies targeting canonical pathways (such as against VEGF, PI3K), IDO inhibitors, TGF-β inhibitors, CXCR2 inhibitors, CXCR4 inhibitors, A2AR inhibitors/anti-CD73
Gut microbiome	Fecal microbiota transplantation, probiotics, diet interventions

ICI: Immune checkpoint inhibitors; TLR: Toll-like receptors; STING: Stimulator of interferon genes; c-di-GMP: Bis-(3′-5′)-cyclic dimeric guanosine monophosphate; JAK: Janus kinase; CTLA-4: Cytotoxic T-lymphocyte-associated protein 4; TIGIT: T cell immunoreceptor with immunoglobulin and ITIM domains; LAG-3: Lymphocyte-activation gene 3; TIM-3: T cell immunoglobulin and mucin-domain containing-3; VISTA: V-type immunoglobulin domain-containing suppressor of T cell activation; CD40: Cluster of differentiation 40; GITR: Glucocorticoid-induced TNFR-related; ICOS: Inducible T cell costimulatory; BTLA: B- and T-lymphocyte attenuator; CSF1R: Colony stimulating factor 1 receptor; VEGF: Vascular endothelial growth factor; PI3K: Phosphoinositide 3-kinases; IDO: indoleamine 2,3-dioxygenase; TGF-β: Transforming growth factor beta; CXCR2: CXC chemokine receptor 2; CXCR4: CXC chemokine receptor 4; A2AR: Adenosine A2A receptor; CD73: Ecto-5′-nucleotidase.
